# A Brief Screening Tool for Opioid Use Disorder: EMPOWER Study Expert Consensus Protocol

**DOI:** 10.3389/fmed.2021.591201

**Published:** 2021-03-31

**Authors:** Dokyoung S. You, Aram S. Mardian, Beth D. Darnall, Chwen-Yuen A. Chen, Korina De Bruyne, Pamela D. Flood, Ming-Chih Kao, Anita D. Karnik, Jennifer McNeely, Joel G. Porter, Robert P. Schwartz, Richard L. Stieg, Sean C. Mackey

**Affiliations:** ^1^Department Anesthesiology, Perioperative and Pain Medicine, Stanford University School of Medicine, Palo Alto, CA, United States; ^2^Department of Family, Community and Preventive Medicine, University of Arizona College of Medicine-Phoenix, Phoenix, AZ, United States; ^3^Phoenix VA Health Care System, Phoenix, AZ, United States; ^4^Division of Primary Care and Population Health, Department of Medicine, Stanford University School of Medicine, Palo Alto, CA, United States; ^5^Division of Primary, Preventive, and Community Care, Stanford University School of Medicine, Palo Alto, CA, United States; ^6^Phoenix VA Health Care System, Department of Psychiatry, University of Arizona College of Medicine-Phoenix, Phoenix, AZ, United States; ^7^Department of Population Health, Section on Tobacco, Alcohol, and Drug Use, New York University School of Medicine, New York, NY, United States; ^8^Intermountain Healthcare, Family Medicine, Layton, UT, United States; ^9^Friends Research Institute, Baltimore, MD, United States; ^10^Private Practitioner, Englewood, CO, United States

**Keywords:** screening tool, opioid use disorder, long-term opioid use, opioid deprescribing, consensus protocol

## Abstract

Growing concerns about the safety of long-term opioid therapy and its uncertain efficacy for non-cancer pain have led to relatively rapid opioid deprescribing in chronic pain patients who have been taking opioid for years. To date, empirically supported processes for safe and effective opioid tapering are lacking. Opioid tapering programs have shown high rates of dropouts and increases in patient distress and suicidal ideation. Therefore, safe strategies for opioid deprescribing that are more likely to succeed are urgently needed. In response to this demand, the EMPOWER study has been launched to examine the effectiveness of behavioral medicine strategies within the context of patient-centered opioid tapering in outpatient settings (https://empower.stanford.edu/). The EMPOWER protocol requires an efficient process for ensuring that collaborative opioid tapering would be offered to the most appropriate patients while identifying patients who should be offered alternate treatment pathways. As a first step, clinicians need a screening tool to identify patients with Opioid Use Disorder (OUD) and to assess for OUD severity. Because such a tool is not available, the study team composed of eight chronic pain and/or addiction experts has extended a validated screening instrument to develop a brief and novel consensus screening tool to identify OUD and assess for OUD severity for treatment stratification. Our screening tool has the potential to assist busy outpatient clinicians to assess OUD among patients receiving long-term opioid therapy for chronic pain.

Growing concerns about adverse effects and potential harms associated with long-term opioid use combined with lack of effectiveness data (1–3) have led national and state organizations to recommend reducing initiation of opioid medications for chronic pain (4, 5), and in some cases to impose mandated tapering (6, 7). To date, empirically supported processes for effective outpatient opioid tapering are lacking, potentially contributing to high rates of patient attrition (~ 53.3%) (8, 9) and seeking other sources of opioids (10). While the literature remains nascent, a pilot study has demonstrated that prescribing physicians can help patients reduce opioid dose by about 50%, without an increase in pain intensity, by using a slow and individualized tapering method at outpatient settings (11). Intensive interdisciplinary programs (12) may be appropriate to reduce opioid medications for patients who have more medical or psychiatric complexity, although access to these programs is notably limited. Furthermore, clinical practice guidelines recommend that prescribing physicians routinely assess patients on long-term opioid therapy for opioid use disorder (OUD) (4). According to the Diagnostic and Statistical Manual of Mental Disorders (DSM)-5, OUD is defined as *a cluster of cognitive, behavioral, and physiological symptoms indicating that the individual continues using the substance despite significant substance-related problems* (13) and exists on a continuum of severity (mild, moderate, and severe) (13). For patients with a diagnosis of OUD, particularly moderate-severe OUD, medication for addiction treatment (MAT) is recommended rather than opioid tapering (14, 15). Therefore, deprescribing approaches need to be tailored to an individual patient to optimize the likelihood of successful reduction.

One challenge in assessing OUD and its severity is that the American Psychiatric Association's DSM has modified various diagnostic terms over the years to define the spectrum of problematic opioid use. Historically, DSM-III (16) and DSM-IV (17) used two distinct diagnostic categories to describe problematic opioid use: *abuse* and *dependence*. The DSM-IV diagnostic term of *abuse* is defined by “a maladaptive pattern of substance use manifested by recurrent and significant adverse consequences related to the repeated use of substances,” and the definition of *dependence* is the same as that of OUD in DSM-5 (13). In contrast to the diagnoses of abuse and dependence in the DSM-IV, the DSM-5 has adopted a dimensional approach (18) in which OUD is now diagnosed along a continuum of mild, moderate and severe. This change requires clinicians to understand both the current and previous diagnostic classification systems as well as the degree of concordance between the two systems because cumulative data for evidence-based addiction treatment have been derived from the previous systems (19, 20) and new treatment studies and recommendations use the current version of DSM. Studies show that patients with DSM-5 moderate or severe OUD have a high degree of concordance with DSM-IV *opioid dependence*. Specifically, DSM-5 moderate to severe OUD yields 99% sensitivity and 99% positive predictive value (PPV) for DSM-IV opioid dependence (21). In contrast, only 18% of patients with mild OUD meet criteria for DSM-IV *opioid dependence* (21). As such, clinicians using a binary diagnostic approach (yes vs. no) for OUD will be limited in offering optimal treatments for diverse patients on long-term opioid therapy. Therefore, accurate and scalable systems to identify OUD and assess for OUD severity are critical for treatment stratification, but no such system or screening tool is currently available (22).

## Development of the EMPOWER Study Expert Consensus Protocol for OUD Screening

The EMPOWER study team first searched for a screening tool to identify OUD by severity categories when preparing to launch a study to investigate the effectiveness of behavioral medicine strategies within the context of patient-centered opioid tapering in outpatient settings (23). Our search yielded several self-report instruments that were validated and widely used to assess prescription opioid misuse and aberrant drug-taking behaviors such as the Current Opioid Misuse Measure (24), Pain Medication Questionnaire (25), and Prescription Opioid Misuse Index (26). There are also self-reports to screen for opioid abuse or dependence such as the NIDA-modified ASSIST (27), Drug Abuse Screening Test (28), Prescription Opioid Abuse Checklist (29), Prescription Drug Use Questionnaire (30), Addiction Behavior Checklist (31), and Rapid Opioid Dependence Screen (32). These existing tools intended for use in patients receiving long-term opioid therapy for chronic pain aim to identify opioid aberrant medication-related behavior, misuse, abuse, or dependence and either suffer from intolerably low sensitivity or are too lengthy for practical use in primary care settings with high patient volumes (33, 34). More importantly, these tools do not provide OUD severity for treatment stratification. After determining the lack of existing suitable tools, the EMPOWER study team decided to develop an efficient and scalable tool to assess OUD severity and identify the most appropriate care pathway.

When launching the EMPOWER study, the study team recognized that opioid tapering is not right for everyone. Patients with moderate or severe symptoms of OUD may be ill-suited to outpatient tapering and require different care pathways (14, 15). To identify OUD and assess OUD severity, the study team identified two candidate tools: the Tobacco, Alcohol, Prescription Medication, and Other Substance use (TAPS-1 and 2) (35) and DSM-5 OUD criteria (13). The TAPS-1 and−2 were developed to screen for a problematic use of alcohol, tobacco, and other substances in patients visiting primary care clinics. The TAPS uses a two-step screening process (35). In the first step, the four-item TAPS-1 is administered to all patients and screens for the use of four substance types in the past 12 months. Then, the appropriate items on TAPS-2 assessing the use of each substance in the past 3 months are administered to those who endorse other than “never” response on the TAPS-1 items. The TAPS has acceptable sensitivity (>0.70) and specificity (>0.85) when screening for problematic use of tobacco, alcohol and marijuana, but unacceptably low sensitivity (0.48) when screening for OUD specific to prescription opioid use (35). Alternatively, clinicians may use the 11 criteria for OUD defined by the DSM-5 OUD (13). Yet, the DSM-5 criteria are rarely used to assess OUD (36) because it is not practical to conduct the clinical interview for every patient on long-term opioid therapy in a busy clinic.

## The EMPOWER Consensus Protocol for OUD Screening and Treatment Stratification

The EMPOWER study team composed of eight chronic pain and/or addiction experts developed a novel consensus protocol to identify OUD and assess for OUD severity in patients taking long-term prescription opioids for chronic pain. The study team modified the two-step process used in the TAPS. To increase sensitivity, the team, in collaboration with the authors of TAPS (35), decided to use all three opioid items in the TAPS-2 as initial screening items, instead of using the TAPS-1. Next, to optimize specificity and assess for OUD severity, the DSM-5 OUD criteria were used in the form of a checklist. Therefore, the EMPOWER consensus protocol for screening and distinguishing severity of OUD includes (Table 1): (Step 1) all three opioid items from the TAPS-2 and (Step 2) the DSM-5 Criteria Checklist for OUD to (Step 3) stratify the severity of OUD. Step 1 can be self-administered in a paper format to all patients on opioid therapy. Based on patients' responses on Step 1, a clinician completes the checklist during the in-person clinical interview only with those endorsing any of the 3 screening items (Step 2).

**Table 1:** The EMPOWER OUD screening protocol.

**STEP 1:**	**Administer the three items from the TAPS-2 Tool:** In the PAST 3 MONTHS,		
	1. Did you use a prescription opioid pain reliever (for example, Percocet, Vicodin) not as prescribed or that was not prescribed for you?	Yes	No
	2. Have you tried and failed to control, cut down or stop using opioid pain relievers?	Yes	No
	3. Has anyone expressed concern about your use of an opioid pain reliever?	Yes	No
**If a patient marks YES to ANY of the 3 items**, administer the Opioid Use Disorder Checklist below			
**STEP 2:**	**DSM-5 Opioid Use Disorder Checklist:** In the PAST 12 MONTHS,		
	1. Opioids are often taken in larger amounts or over a longer period than was intended.	Yes	No
	2. There is a persistent desire or unsuccessful efforts to cut down or control opioid use.	Yes	No
	3. A great deal of time is spent in activities necessary to obtain the opioid, use the opioid, or recover from its effects.	Yes	No
	4. Craving, or a strong desire or urge to use opioids.	Yes	No
	5. Recurrent opioid use resulting in a failure to fulfill major role obligations at work, school, or home.	Yes	No
	6. Continued opioid use despite having persistent or recurrent social or interpersonal problems caused or exacerbated by the effects of opioids.	Yes	No
	7. Important social, occupational, or recreational activities are given up or reduced because of opioid use.	Yes	No
	8. Recurrent opioid use in situations in which it is physically hazardous.	Yes	No
	9. Continued opioid use despite knowledge of having a persistent or recurrent physical or psychological problem that is likely to have been caused or exacerbated by the substance.	Yes	No
	10. Tolerance, as defined by either of the following: • A need for markedly increased amounts of opioids to achieve intoxication or desired effect. • A markedly diminished effect with continued use of the same amount of an opioid.		
	**(Note: This criterion is not considered to be met for those taking opioids solely under appropriate medical supervision.)**	Yes	No
	11. Withdrawal, as manifested by either of the following: • The characteristic opioid withdrawal syndrome. • Opioids (or a closely related substance) are taken to relieve or avoid withdrawal symptoms.		
	**(Note: This criterion is not considered to be met for those individuals taking opioids solely under appropriate medical supervision.)**	Yes	No
	Sum the number of symptoms indicated above: SUM SCORE:	—————-	
**STEP 3:**	**Interpretation of DSM-5 Opioid Use Disorder Checklist Results**		
	**No OUD**: Presence of 0-1 symptom.		
	• OK for outpatient opioid tapering		
	**Mild**: Presence of 2–3 symptoms. 305.50 (F11.10)		
	• OK to consider for outpatient opioid tapering, or intensive rehabilitation program for opioid tapering, or referral for OUD treatment		
	**Moderate**: Presence of 4–5 symptoms. 304.00 (F11.20)		
	• Referral for OUD treatment		
	**Severe**: Presence of 6 or more symptoms. 304.00 (F11.20)		
	• Referral for OUD treatment		

*For the EMPOWER study, STEP 2 items are adapted into questions*.

The opioid-prescribing clinician has historical knowledge of the opioid and other drug taking behavior of their patients; this, combined with the medical record and prescription drug monitoring databases, can serve as collateral information in determining a diagnosis and its corresponding severity. Clinicians are reminded of the caveat for tolerance and withdrawal symptoms in Table 1, Step 2. These two underlined items are not considered criteria for OUD when opioids are taken as prescribed; rather, tolerance and withdrawal symptoms are indices of expected normal physiologic adaptation to persistent opioid use, which is distinct from addiction or opioid use disorder.

## Strength and Limitations of the EMPOWER Study Expert Consensus Protocol

In the absence of existing validated methods, we provide our consensus screening protocol in response to the urgent societal need for such tools so that (a) clinicians may offer appropriate treatments to a heterogeneous patient population receiving long-term opioid therapy for chronic pain (Table 1, Step 3), and (b) researchers can study characteristics and treatment outcomes of patients with appropriate opioid use and across the spectrum of OUD severity. Our screening protocol can also facilitate efficient evaluation of eligibility for our multi-site pragmatic study of patient-centered opioid tapering (23). We are currently investigating the validity and reliability of administrating our protocol in an electronic format using an open-source learning healthcare system (https://choir.stanford.edu/). Additionally, we will examine data for patients who are screened as having no or mild OUD severity using our two-step protocol and will characterize their opioid taper and pain reduction outcomes.

Achieving optimal results for prescription opioid tapering requires careful assessment of patients and their opioid-taking behaviors because such assessment ensures that effective individualized opioid tapering or opioid addiction treatments are delivered to patients according to their individual needs. The EMPOWER study team found a gap in existing approaches for practical, individualized opioid deprescribing due to the lack of a brief tool to identify OUD and stratify severity for patients with chronic pain receiving long-term opioid therapy. Our study team has developed a streamlined consensus protocol to identify OUD and assess for OUD severity for clinical and research use.

## Data Availability Statement

The original contributions presented in the study are included in the article/supplementary material, further inquiries can be directed to the corresponding author/s.

## Author Contributions

DY: conceptualization, writing—original draft, review, and editing. AM: conceptualization, methodology, writing—original draft, and review and editing. BD: conceptualization, methodology, writing—review and editing, and project administration. C-YC, KD, PF, AK, JM, JP, RPS, and RLS: conceptualization, methodology, writing—review and editing. M-CK: conceptualization and methodology. SM: conceptualization, methodology, writing—review and editing, project administration, and supervision. All authors: contributed to the article and approved the submitted version.

## Conflict of Interest

RPS reports serving as a consultant to Verily Life Sciences, Ltd. The remaining authors declare that the research was conducted in the absence of any commercial or financial relationships that could be construed as a potential conflict of interest.
